# Intraoperative Optical Coherence Tomography Using the RESCAN 700: Preliminary Results in Collagen Crosslinking

**DOI:** 10.1155/2015/572698

**Published:** 2015-04-06

**Authors:** Natasha Pahuja, Rohit Shetty, Chaitra Jayadev, Rudy Nuijts, Bharath Hedge, Vishal Arora

**Affiliations:** ^1^Department of Cornea & Refractive Surgery, Narayana Nethralaya 121/C, Chord Road, 1st “R” Block, Rajajinagar, Bangalore, Karnataka 560 010, India; ^2^Department of Ophthalmology, Maastricht University Medical Center, Netherlands; ^3^Forus Health Pvt. Ltd, No. 2234, 23rd Cross, Banashankari 2nd Stage, Bangalore 560070, India; ^4^Department of Cataract & Refractive Lens Surgery, Narayana Nethralaya, 121/C, Chord Road, 1st “R” Block Rajajinagar, Bangalore, Karnataka 560 010, India

## Abstract

*Purpose*. To compare the penetration of riboflavin using a microscope-integrated real time spectral domain optical coherence tomography (ZEISS OPMI LUMERA 700 and ZEISS RESCAN 700) in keratoconus patients undergoing accelerated collagen crosslinking (ACXL) between epithelium on (epi-on) and epithelium off (epi-off). *Methods*. Intraoperative images were obtained during each of the procedures. Seven keratoconus patients underwent epi-on ACXL and four underwent epi-off ACXL. A software tool was developed using Microsoft.NET and Open Computer Vision (OpenCV) libraries for image analysis. Pre- and postprocedure images were analyzed for changes in the corneal hyperreflectance pattern as a measure of the depth of riboflavin penetration. *Results*. The mean corneal hyperreflectance in the epi-on group was 12.97 ± 1.49 gray scale units (GSU) before instillation of riboflavin and 14.46 ± 2.09 GSU after AXCL (*P* = 0.019) while in the epi-off group it was 11.43 ± 2.68 GSU and 16.98 ± 8.49 GSU, respectively (*P* = 0.002). The average depth of the band of hyperreflectance in the epi-on group was 149.39 ± 15.63 microns and in the epi-off group it was 191.04 ± 32.18 microns. *Conclusion*. This novel *in vivo*, real time imaging study demonstrates riboflavin penetration during epi-on and epi-off ACXL.

## 1. Introduction

Keratoconus is a bilateral, asymmetric, degenerative disorder of the cornea that is characterized by progressive thinning of the stroma, leading to significant visual morbidity. Though the disease has been studied extensively in the past, its etiology is still unclear [1–3]. Of the various treatment options the most effective in delaying its progression is corneal collagen crosslinking (CXL) [4, 5]. The surgical technique of standard crosslinking involves removal of the epithelium to allow penetration of riboflavin into the stroma and then irradiation with ultraviolet A (UVA) light. There is still no consensus on how long the stroma should be imbibed with the solution (riboflavin 0.1% and dextran T 500 20%) in order to ensure a sufficient intrastromal concentration of vitamin B2. While some groups have studied the depth of riboflavin penetration [6, 7], others have deliberated on the importance of removal of corneal epithelium during CXL [8, 9]. Use of imaging modalities like the optical coherence tomography (OCT), Brillouin microscopy, and second harmonic imaging has allowed better understanding of changes occurring in the cornea and thereby helps in predicting treatment outcomes [10–13]. These methods, however, do not allow the assessment of tissues* in vivo*. We have previously compared the extent of penetration of riboflavin in epi-on and epi-off procedures with a hand held spectral domain OCT (SD-OCT, Bioptigen, Inc.) with few limitations [7].

The microscope integrated intraoperative SD-OCT is a technology that offers continuous real time visualization of the corneal changes* in vivo* during surgical procedures. The surgical microscope (OPMI LUMERA 700, Zeiss) is combined with an SD OCT having a wavelength of 840 nm and scanning speed of 27000 A-scans per second (RESCAN 700 from ZEISS). We used this system to observe the depth of penetration of riboflavin as well as to compare the difference in the reflectance pattern of the cornea in real time between accelerated collagen crosslinking (ACXL) without debridement of epithelium (epi-on) and ACXL with epithelium debrided (epi-off).

## 2. Materials and Methods

This observation study was conducted at a tertiary care center in Bangalore, India. The study protocol was approved by the institute's ethics committee and was conducted with strict adherence to the guidelines laid down by the declaration of Helsinki. The study comprised 11 eyes of 11 consecutive patients: eight males and three females. The mean age was 20 years (14 to 26 years). Details of all patients are given in Table 1.

All patients underwent preoperative visual acuity testing and complete slit lamp examination. Progression of keratoconus was defined as an increase of 0.5 diopter (*D*) or more in two or more keratometry values in the steep meridian between two sagittal curve maps or a decrease in corneal thickness of 10% or more at the thinnest point between two pachymetry maps on Pentacam (Oculus, Wetzlar, Germany) in the preceding 6 months [1, 2]. Seven patients who had a corneal thickness of <420 *μ*m underwent epi-on ACXL. However, in patient 1 (age 14 years) and patient 5 (age 15 years), due to their younger age group, epi-on ACXL was preferred even with a pachymetry ≥ 420 *μ*m for better postoperative comfort. After anesthetizing the eye with topical proparacaine 0.5% (Paracaine, Sunways, (India) Pvt. Ltd), 0.25% riboflavin solution containing hydroxypropyl methylcellulose and benzalkonium chloride (ParaCel, Avedro Inc. USA) was applied every 90 seconds for a total of 4 minutes followed by an application of 0.22% riboflavin isotonic solution every 90 seconds for a total of six minutes (VibeX Xtra, Avedro Inc. USA). After rinsing the cornea with balanced salt solution (BSS), ultraviolet A (UV-A) irradiation was initiated using 45 mW/cm^2^ for 2 minutes and 40 seconds for a surface dose of 7.2 J [14].

Four patients with adequate corneal thickness underwent ACXL after corneal epithelial debridement. The central 7–9 mm of the epithelium was removed with a mechanical epithelium scraper followed by application of 0.1% riboflavin 5-phosphate and 20% dextran solution once every 2 minutes for 20 minutes. Exposure to UV-A (30 mW/cm^2^) radiation for 4 minutes was done in all these. A soft bandage contact lens (BCL) with good oxygen permeability was placed at the end of the procedure. The patients were reviewed till the removal of the BCL.

For intraoperative imaging we used the RESCAN 700 (Zeiss), which is a real time intraoperative SDOCT integrated with the operating microscope. This system is based on the fiber optics Michelson interferometer configuration that allows noninvasive tissue observation through sectional cuts of the ocular structure in real time. The scan depth is 2 mm and scan length can vary from 3 to 16 mm. The axial and transverse resolution of the anterior segment OCT in tissue are 5 *μ*m and 15.5 *μ*m, respectively. The microscope provides uninterrupted OCT imaging and video recording during the entire length of the surgery in a small window adjacent to the operating field. The OCT images are recorded in a horizontal and vertical orientation. The RESCAN 700 includes Z-tracking and focus control for image stabilization and quality control. Due to the possibility of inactivation of riboflavin due to light, only intermittent OCT scanning was done during the procedure. The video output was stored and later reviewed for critical steps of the surgery. A single observer performed all the image acquisitions with enhanced image quality. A cube (512 × 128) was used to image the required area. The size of the cube was adjusted in order to achieve optimal coverage.

A software tool was developed separately using Microsoft.NET and Open Computer Vision (OpenCV) libraries for image analysis. Images were first extracted from the video sequence. The region of interest was automatically marked using polynomial regression (curve fitting, 5th order) with an option for fine adjustment of the selected region. The boundary of the selected region could be altered using a tool similar to the pen tool of Adobe Photoshop. Once the region of interest (ROI) was marked, a sliding window approach was used to measure the parameters within the ROI. A window of dimension 10× was used and the average intensity was calculated by summing up the intensity values and dividing it by the number of pixels (10 × ROI height). A histogram was generated on the active window region and based on the mode (most repeated value, peak of the histogram) a threshold was determined. When this threshold was applied to the windowed region, pixels with less gray values were removed and only the hyperluminescent (reflective) ones were retained. To identify the presence of hyperluminescence, the image of the corneal cross section was divided into the anterior two-thirds and posterior one-third. The ratios of average intensities were calculated for images before instillation of riboflavin and at the end of the procedure. The images were enhanced using contrast stretching and the software measured the dimensions (depth) of hyperluminescence/reflectance. The first image taken prior to starting instillation of riboflavin served as a control with any prior hyperreflectivity inherent to the corneal stroma taken as baseline. The second image was taken at the end of the entire CXL procedure. The band of increased reflectivity seen in the corneal stroma after the procedure was taken as a representation of the penetration of riboflavin. The extracted data were correlated and analyzed. Finally the scale was converted from pixels to microns using the data sheet provided by the manufacturer.

### 2.1. Statistical Analysis

The OCT images were analyzed and the data thus obtained was entered into an Excel spreadsheet (Microsoft Corp.). Statistical analysis of the results was performed using the SPSS software (version 22, IBM SPSS Statistics). Normality of the distribution of all measurements was confirmed using the Shapiro-Wilk test, which is more appropriate for small sample sizes than the Kolmogorov-Smirnov test. Student's *t*-test for paired data was used; a *P* value of less than 0.05 was considered statistically significant. The mean of the corneal stromal hyperreflectance and ratio of reflectance of the anterior two-thirds and posterior one-third of the cornea in each group was used for the statistical analysis.

## 3. Results

**Figure 1:**
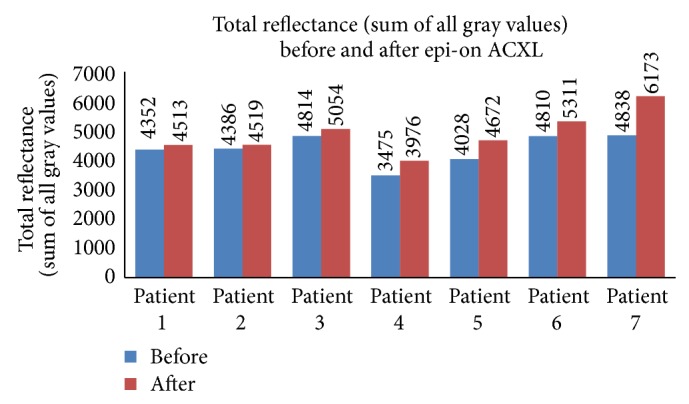
Total reflectance (sum of all gray values) seen in patients undergoing epi-on ACXL before and after the procedure.

**Figure 2:**
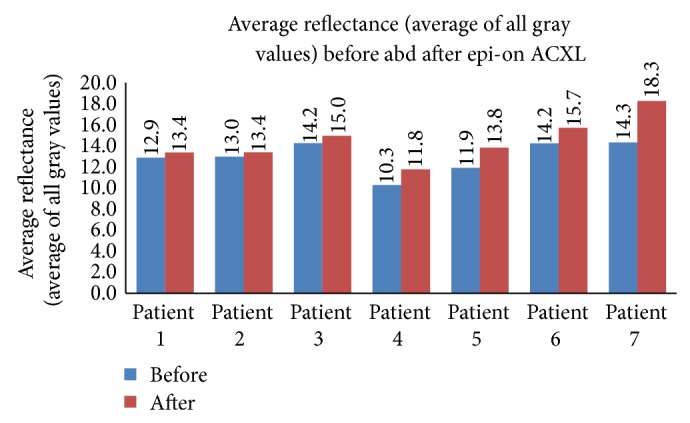
Average reflectance (average of all gray values) seen in patients undergoing epi-on ACXL before and after the procedure.

**Figure 3:**
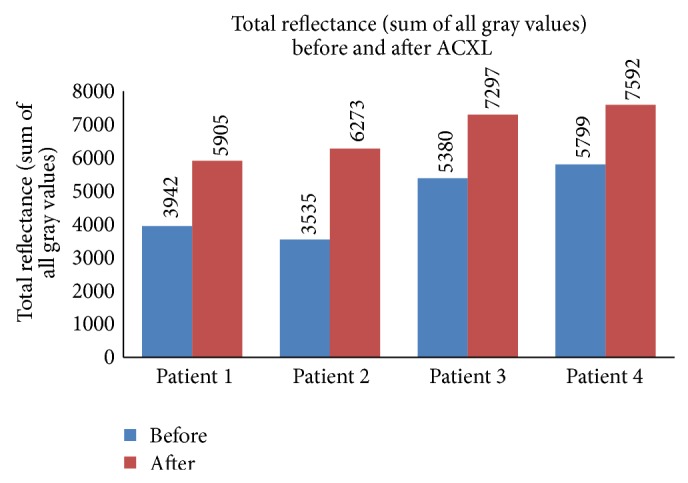
Total reflectance (sum of all gray values) seen in patients undergoing epi-off ACXL before and after the procedure.

**Figure 4:**
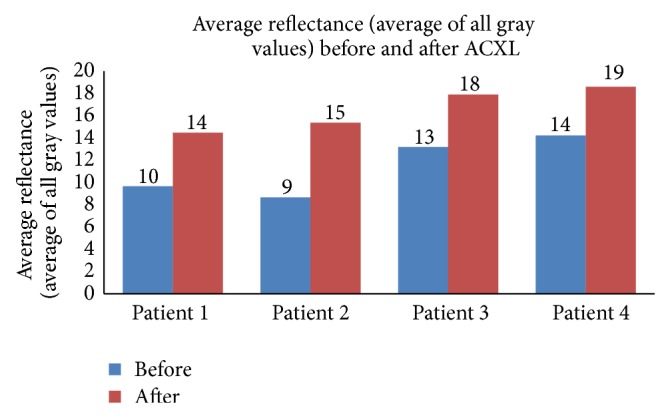
Average reflectance (average of all gray values) seen in patients undergoing epi-off ACXL before and after the procedure.

The total reflectance (sum of all gray values) in the epi-on group (Figure 1) was 4386.13 ± 497.38 gray scale units (GSU) before ACXL and 4888.03 ± 387.62 GSU after ACXL and the average reflectance (Figure 2) was 12.97 ± 1.49 GSU before starting the procedure and 14.46 ± 2.09 GSU after ACXL (*P* = 0.019). The total reflectance in the epi-off group (Figure 3) was 4664.0 ± 1094.88 GSU and 6767.0 ± 805.74 GSU before and after the procedure, respectively. The mean corneal hyperreflectance (Figure 4) in the same group was 11.43 ± 2.68 GSU and 16.98 ± 8.49 GSU before and after ACXL, respectively (*P* = 0.002). The mean of the ratio of the anterior two-thirds and posterior one-third of cornea in epi-on group before starting of the procedure was 51.95 ± 10.22 GSU and after ACXL was 57.34 ± 10.23 GSU (*P* = 0.035) and in the epi-off group it was 44.63 ± 16.98 GSU and 64.63 ± 7.21 GSU before and at the end ACXL, respectively (*P* = 0.034).

**Figure 5:**
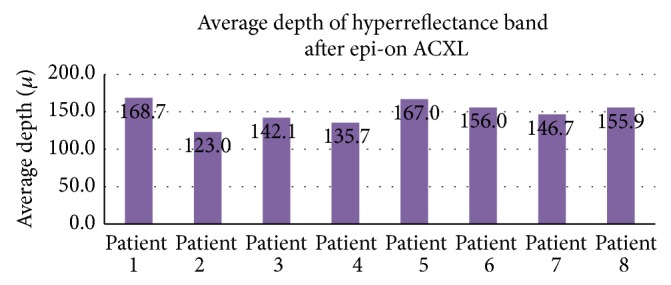
Average depth of hyperreflectance band seen in patients after epi-on ACXL.

**Figure 6:**
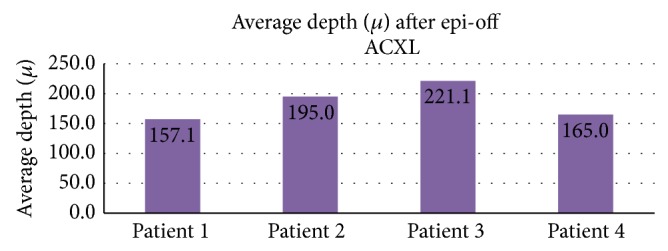
Average depth of hyperreflectance band seen in patients after epi-off ACXL.

The average depth of the hyperreflective band in the epi-on group was 149.39 ± 15.63 microns (Figure 5) and in the epi-off group was 191.04 ± 32.18 microns (Figure 6).

## 4. Discussion 

Corneal collagen crosslinking is currently the most preferred treatment option for the management of progressive keratoconus and other corneal ectasias [3, 4] and works by increasing the biomechanical stability of the cornea [6, 15]. There are several CXL protocols using different energy levels with and without epithelial debridement [14, 16–18]. Without epithelial debridement the major theoretical challenge is in the penetration of the hydrophilic macromolecule of riboflavin across the hydrophobic corneal epithelial barrier. With continued research on the safety and efficacy of transepithelial CXL (TECXL) [14] and use of drug delivery systems like iontophoresis to achieve riboflavin delivery into the cornea [19], TECXL is fast emerging as a treatment option for patients with thin corneas giving encouraging results [17].

It is therefore interesting to know the depth of riboflavin in the various protocols of CXL. Researchers have used keratocyte apoptosis after corneal collagen crosslinking as an indirect measure of depth and effectiveness of this procedure [20]. Seiler and Hafezi have used SD-OCT as an effective biomicroscopic tool to determine the depth of riboflavin penetration [21]. They have assessed the effectiveness of the procedure with the help of a demarcation line noticed two weeks after CXL. Caporossi et al. performed confocal microscopy analyses in humans after crosslinking and detected* in vivo* the effective depth of treatment by identifying distinct vertical and lateral transition areas at a depth of 270 to 330 *μ*m [22].

**Figure 7:**
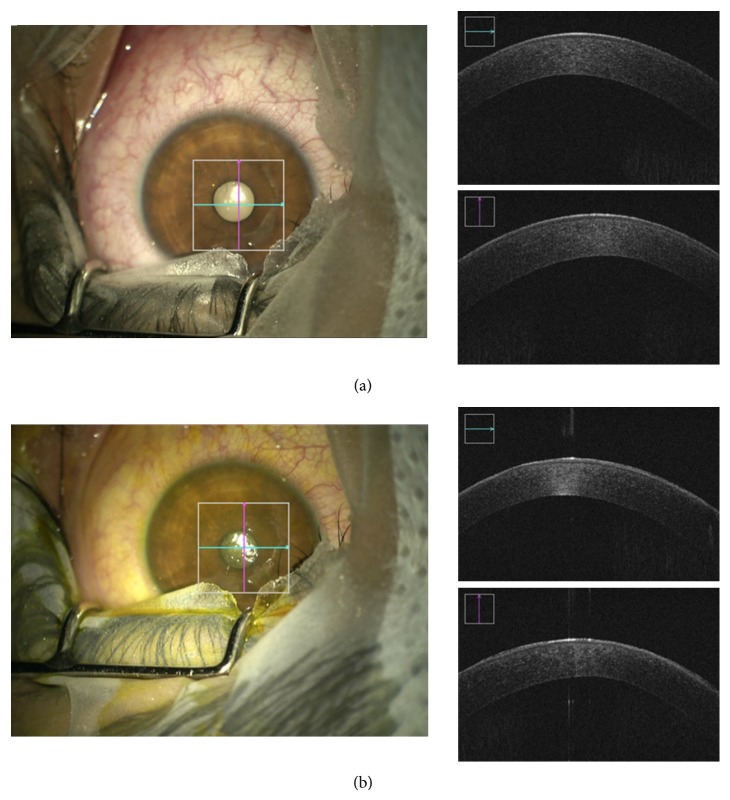
Epi-on ACXL being performed under ZEISS OPMI LUMERA 700. (a) Preprocedure picture with intraoperative OCT (top right); (b) postprocedure picture with intraoperative OCT (bottom right).

**Figure 8:**
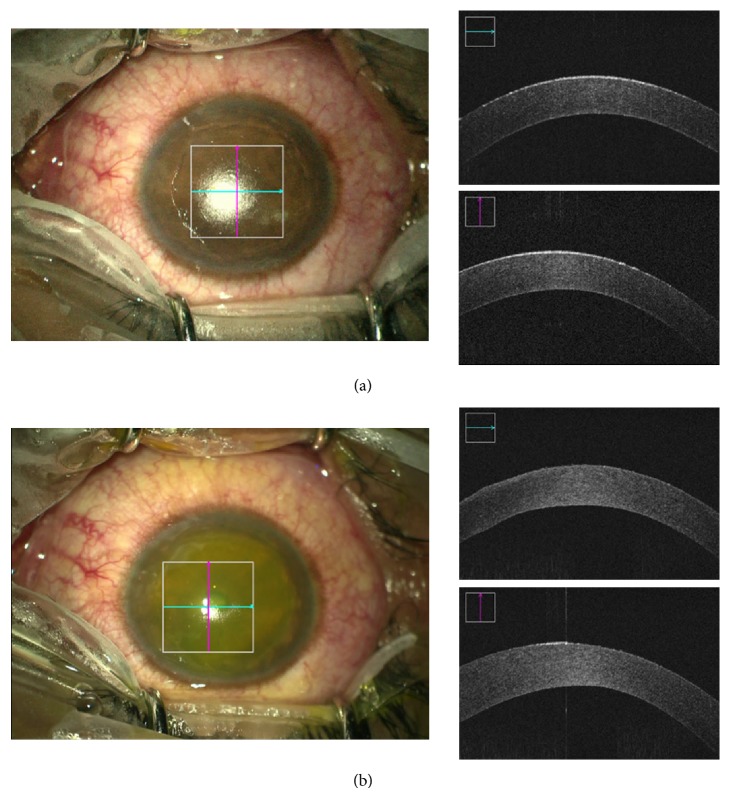
Epi-off ACXL being performed under ZEISS OPMI LUMERA 700. (a) Preprocedure picture with intraoperative OCT (top right); (b) postprocedure picture with intraoperative OCT (bottom right).

John Kanellopoulos and Asimellis introduced a novel, noninvasive, quantitative technique utilizing anterior segment OCT images to quantitatively assess the depth and cross-sectional area of CXL in the corneal stroma based on digital image analysis [23]. Our study is comparable in that the SDOCT was used to image the depth, but we used microscope integrated real time* in vivo* imaging. We used the change in reflectance pattern depth as a measure of riboflavin penetration by taking images before and after the procedure. We also compared the difference in riboflavin penetration between epithelium debrided and transepithelial crosslinking. Hence in this study, using the intraoperative SD-OCT, we describe a zone of hyperreflectance as a measure of the depth of penetration of riboflavin (Figures 7 and 8).

We have previously used the hand held SD-OCT to show the penetration of riboflavin for both epithelium-on and epithelium-off procedures [7]. However, since it was not integrated into the operative microscope, image acquisition was cumbersome. Also due to the lack of continuous video capturing mode, serial image capture throughout the procedure was not possible. This was overcome with the use of an intraoperative real time OCT, with serial image acquisition. In the previous study we found that the mean depth of the hyperreflective band after epi-off CXL was 54.2 ± 5.2 *μ*m and 72.4 ± 7.1 *μ*m at 30 and 60 minutes, respectively. In this study, the average depth of the hyperreflective band in the epi-on group was 149.39 ± 15.63 microns and in the epi-off group was 191.04 ± 32.18 microns. This difference could be attributed to the different devices used for imaging, the method of image analysis, and the different CXL protocols used.

There was an increase in the depth of reflectance in both crosslinking groups after the procedure suggestive of penetration of the drug into the anterior corneal stroma. Since the second image was acquired after CXL, its influence on the reflectance pattern is a strong possibility. Nevertheless, this imaging technology can be used to possibly compare the effect of crosslinking in various protocols, in riboflavin concentrations, in dyes and energy settings, in real time, intraoperatively, and* in vivo *and hence help refining protocols for CXL. To the best of our knowledge, this study is the first to assess quantitatively the penetration of riboflavin during CXL* in vivo*, in both epithelium-on and epithelium-off procedures using the microscope integrated intraoperative SD OCT.

## 5. Conclusion

This novel* in vivo* imaging study demonstrates the penetration of riboflavin during ACXL in both epithelium-on and epithelium-off procedures. The hyperreflectance noted in the images shows a penetration of approximately 149.39 ± 15.63 microns and 191.04 ± 32.18 microns in epi-on and epi-off groups, respectively. Long-term postoperative evaluation of changes in corneal biomechanics will further help validate our findings.

## Figures and Tables

**Table 1:** This table shows the mean keratometry (Km), mean thinnest cornea thickness (TCT), mean refractive spherical equivalent (MRSE), and grade of keratoconus as per the Amsler-Krumeich classification grade of patients enrolled in the study.

	Km (*D*)	TCT (micron)	MRSE	Grade of keratoconus
Patient 1 (epi-on)	54	429	−4.5	2
Patient 2 (epi-on)	48.6	408	−6.25	2
Patient 3 (epi-on)	62.1	417	−16	4
Patient 4 (epi-on)	54.9	415	−13	4
Patient 5 (epi-on)	46.7	420	−2.5	1
Patient 6 (epi-on)	48.1	411	−5	2
Patient 7 (epi-on)	54.3	390	−4	3
Patient 1 (epi-off)	47.1	496	−4.5	1
Patient 2 (epi-off)	43.5	490	−2.5	1
Patient 3 (epi-off)	47.9	485	−2	1
Patient 4 (epi-off)	48.2	472	−1	2

## References

[1] Rabinowitz Y. S. (1998). Keratoconus. *Survey of Ophthalmology*.

[2] Krachmer H., Mannis M. J., Holland E. J. (2011). *Cornea*.

[3] Jeyabalan N., Shetty R., Ghosh A., Anandula V. R., Ghosh A. S., Kumaramanickavel G. (2013). Genetic and genomic perspective to understand the molecular pathogenesis of keratoconus. *Indian Journal of Ophthalmology*.

[4] Wollensak G., Spoerl E., Seiler T. (2003). Riboflavin/ultraviolet-a-induced collagen crosslinking for the treatment of keratoconus. *American Journal of Ophthalmology*.

[5] Shetty R. (2013). Keratoconus and corneal collagen cross-linking. *Indian Journal of Ophthalmology*.

[6] Kymionis G. D., Tsoulnaras K. I., Grentzelos M. A. (2014). Corneal stroma demarcation line after standard and high-intensity collagen crosslinking determined with anterior segment optical coherence tomography. *Journal of Cataract & Refractive Surgery*.

[7] Malhotra C., Shetty R., Kumar R. S., Veluri H., Nagaraj H., Shetty K. B. (2012). In vivo imaging of riboflavin penetration during collagen cross-linking with hand-held spectral domain optical coherence tomography. *Journal of Refractive Surgery*.

[8] Khairy H. A., Marey H. M., Ellakwa A. F. (2014). Epithelium-on corneal cross-linking treatment of progressive keratoconus: a prospective, consecutive study. *Clinical Ophthalmology*.

[9] Kocak I., Aydin A., Kaya F., Koc H. (2014). Comparison of transepithelial corneal collagen crosslinking with epithelium-off crosslinking in progressive keratoconus. *Journal Francais d'Ophtalmologie*.

[10] Mazzotta C., Caragiuli S. (2014). Intraoperative corneal thickness measurement by optical coherence tomography in keratoconic patients undergoing corneal collagen cross-linking. *The American Journal of Ophthalmology*.

[11] Rocha K. M., Perez-Straziota C. E., Stulting R. D., Randleman J. B. (2014). Epithelial and stromal remodeling after corneal collagen cross-linking evaluated by spectral-domain OCT. *Journal of Refractive Surgery*.

[12] Scarcelli G., Kling S., Quijano E., Pineda R., Marcos S., Yun S. H. (2013). Brillouin microscopy of collagen crosslinking: noncontact depth-dependent analysis of corneal elastic modulus. *Investigative Ophthalmology and Visual Science*.

[13] Matteini P., Ratto F., Rossi F. (2009). Photothermally-induced disordered patterns of corneal collagen revealed by SHG imaging. *Optics Express*.

[14] Taneri S., Oehler S., Lytle G., Dick H. B. (2014). Evaluation of epithelial integrity with various Transepithelial corneal cross-linking protocols for treatment of keratoconus. *Journal of Ophthalmology*.

[15] Kanellopoulos A. J. (2009). Collagen cross-linking in early keratoconus with riboflavin in a femtosecond laser-created pocket: initial clinical results. *Journal of Refractive Surgery*.

[16] Mrochen M. (2013). Current status of accelerated corneal cross-linking. *Indian Journal of Ophthalmology*.

[17] Hafezi F., Mrochen M., Iseli H. P., Seiler T. (2009). Collagen crosslinking with ultraviolet-A and hypoosmolar riboflavin solution in thin corneas. *Journal of Cataract and Refractive Surgery*.

[18] Cummings A. B., McQuaid R., Mrochen M. (2013). Newer protocols and future in collagen cross-linking. *Indian Journal of Ophthalmology*.

[19] Arboleda A., Kowalczuk L., Savoldelli M. (2014). Evaluating in vivo delivery of riboflavin with Coulomb-controlled iontophoresis for corneal collagen cross-linking: a pilot study. *Investigative Ophthalmology & Visual Science*.

[20] Wollensak G., Spoerl E., Wilsch M., Seiler T. (2004). Keratocyte apoptosis after corneal collagen cross-linking using riboflavin/UVA treatment. *Cornea*.

[21] Seiler T., Hafezi F. (2006). Corneal cross-linking-induced stromal demarcation line. *Cornea*.

[22] Caporossi A., Mazzotta C., Baiocchi S., Caporossi T., Paradiso A. L. (2012). Transepithelial corneal collagen crosslinking for keratoconus: qualitative investigation by in vivo HRT II confocal analysis. *European Journal of Ophthalmology*.

[23] John Kanellopoulos A., Asimellis G. (2013). Introduction of quantitative and qualitative cornea optical coherence tomography findings induced by collagen cross-linking for keratoconus: a novel effect measurement benchmark. *Clinical Ophthalmology*.

